# Differences in fine-scale spatial genetic structure across the distribution range of the distylous forest herb *Pulmonaria officinalis* (Boraginaceae)

**DOI:** 10.1186/1471-2156-14-101

**Published:** 2013-10-18

**Authors:** Sofie Meeus, Olivier Honnay, Hans Jacquemyn

**Affiliations:** 1 Plant Conservation and Population Biology, Biology Department, University of Leuven, Kasteelpark Arenberg 31, Heverlee, 3001, Belgium

**Keywords:** Fine-scale spatial genetic structure, Disjunct, Core, Mating system, Morph clustering, Spatial autocorrelation

## Abstract

**Background:**

Geographical ranges of plants and their pollinators do not always entirely overlap and it has been suggested that the absence of specialized pollinators at range margins may induce changes in mating systems. Because a species’ mating system is known to have a considerable effect on within-population pollen movement, the extent of fine-scale spatial genetic structure (SGS) can be expected to differ between populations located at different parts of their geographical range. To test this prediction, we compared the fine-scale SGS between two core and two disjunct populations of the distylous forest herb *Pulmonaria officinalis*. Because in disjunct populations of this species the heteromorphic self-incompatibility system showed relaxation in the long-styled morph, but not in the short-styled morph, we also hypothesized that the extent of fine-scale SGS and clustering differed between morphs.

**Results:**

Spatial autocorrelation analyses showed a significant decrease in genetic relatedness with spatial distance for both core and disjunct populations with the weakest SGS found in one of the core populations (*Sp* = 0.0014). No evidence of stronger SGS in the long-styled morph was found in the center of the range whereas one disjunct population showed a significantly (*P* = 0.029) higher SGS in the long-styled morph (*Sp*_L_ = 0.0070) than in the short-styled morph (*Sp*_S_ = 0.0044).

**Conclusions:**

Consistent with previous analyses on distylous plant species, we found weak, but significant spatial genetic structure. However, the extent of SGS varied substantially between populations within regions, suggesting that population characteristics other than mating system (e.g. local pollinator assemblages, population history) may be as important in determining variation in SGS.

## Background

In natural plant populations, genetic diversity and the extent of spatial genetic structure are determined by a variety of population characteristics, ecological conditions and historical events that affect natural selection, gene flow and genetic drift [1-3]. Population characteristics such as size, density and spatial isolation are largely dependent on the geographical location of the population within a species’ range [4,5]. Populations at range edges or populations that are disconnected from the main distribution area of a species are often smaller, less dense, and more isolated than those in the center of the distribution range [3,5,6].

Small and isolated plant populations may be less conspicuous for pollinators, leading to fewer visits, changes in pollinator behavior and decreased reproductive success [7-11]. Moreover, distribution ranges of plant and insect pollinator species sometimes do not entirely overlap, which further affects the efficiency of pollen transfer, especially in plant species with highly specialized breeding systems that require specialist pollinators for precise pollen deposition [12-17]. As a result of altered or incomplete pollinator communities, populations occurring at range margins often suffer more from cross pollen limitation than populations in the center of the distribution area (e.g. *Lupinus perennis*, *Clarkia xantiana*, *Embothrium coccineum*) [10,18,19].

To counterbalance the reduced seed set resulting from pollinator and pollen limitation at range margins, many species have adapted through changes in floral morphology, development and/or physiology that facilitate selfing in order to maintain a certain level of reproduction in pollinator poor environments [15,20-23]. In heterostylous species that are characterized by a heteromorphic incompatibility system and the co-occurrence of at least two different style morphs, breakdown of the breeding system and changes in floral traits have been observed after colonization of disjunct regions such as islands that lack specialized pollinators [23,24]. Natural selection for reproductive assurance in these pollinator poor regions might favor a modified morph capable of self-fertilization, resulting in asymmetrical mating between morphs, biased population morph ratios and eventually breakdown of the heterostylous syndrome [25-29]. The transition from dimorphic to monomorphic populations of *Eichhornia paniculata*, for example, involved the reproductive advantage of a modified, autogamous M-morph compared to the outcrossing L-morph in small ephemeral populations on Jamaica with a low frequency of (long-tongued) pollinators [24].

Changes in floral traits and the associated mating system may have a large impact on the extent of fine-scale spatial genetic structure (SGS) in a population. Under spatially and temporally homogeneous abiotic conditions, SGS results from restricted gene flow through seed and/or pollen dispersal within the population [30,31]. The strength and extent of SGS therefore depends strongly on population characteristics and life-history traits such as plant density, pollinator community and the plant’s mating system, all affecting the relationship between genetic relatedness among individuals and their spatial distance (reviewed in [1,32]). A significantly stronger spatial genetic structure in selfing species compared to outcrossing or self-incompatible species has for example been found by Vekemans and Hardy [32] through the reanalysis of data from 47 plant species. In addition, high densities of individuals decreased the strength of SGS, due to increasing overlap of seed shadows [32]. Although the extent of spatial genetic structure is generally weak in heterostylous species, due to the heteromorphic self-incompatibility system that promotes mating between individuals of the opposite morph (disassortative / intermorph mating), changes in the mating system across the distribution range of heterostylous species can be expected to have an impact on the extent of their fine-scale population genetic structure.

To test the hypothesis that geographical location influences the clustering of related individuals within the population, we compared the extent of SGS in different populations across the distributional range of the distylous forest herb *Pulmonaria officinalis*. Previous research has shown relaxation of the self-incompatibility system in the long-styled morph, but not in the short-styled morph (hereafter L-morph and S-morph, respectively) in western Belgium, where it grows in the disjunct range of this species [33]. In contrast, Hildebrand [34] reported strict self-incompatibility in populations of *P. officinalis* in the core of its distribution range. Investigation of pollination patterns within disjunct populations further showed that both the total stigmatic pollen load and the proportion of legitimate pollen decreased with increasing spatial isolation [35]. Legitimate (intermorph) pollen transfer was, however, asymmetric and decreased more rapidly with decreasing proximity to a compatible legitimate mating partner in the S-morph than in the L-morph. These findings thus indicate that the spatial distribution of potential mates causes morph-specific differences in pollen deposition rates and female reproductive success. We therefore also tested the hypothesis that differences in mating between morphs will manifest themselves in differences in SGS between morphs and in the degree of spatial clustering, and that this effect will be most pronounced in populations in the disjunct range where self-incompatibility has been shown to be leaky in the L-morph.

## Methods

### Study species

*P. officinalis* L. (Boraginaceae) or common lungwort is a herbaceous, autotetraploid, semi-evergreen perennial plant species that grows in the understorey of European species-rich mixed and open forests on relatively humid, wet and loamy soils [36-39]. Populations of *P. officinalis* can be found throughout most of central Europe, occurring from southern Sweden and Denmark in the north to Italy and Bulgaria in the south [36,40]. Outside its main Central-European distribution range, beyond the western edge of the range, fragmented *P. officinalis* populations are found in Belgium as well as in Britain [40,41]. Here, the species became naturalized, presumably after its introduction during medieval times as a medicinal plant [29,42,43].

*P. officinalis* flowers in early spring prior to complete closure of the forest canopy. *P. officinalis* is a distylous species, implying that two style morphs, a short- and a long-styled morph, coexist in one population. Flowers are mainly pollinated by generalist insect species with short proboscises, including *Bombus terrestris*, *B. pascuorum*, *B. pratorum* and *Bombylius major*, whereas in the center of its distribution range (Central Europe) flowers are mainly pollinated by specialist *Anthophora* species with long proboscises [33,44]. Flowers contain only four ovules per flower as in most species of the Boraginaceae. Seeds (3–5 mm) ripen from May until June and are provided with an elaiosome, a nutritious structure attached to the seed (or fruit) that facilitates dispersal by ants [45]. Clonal propagation by woody rhizomes forms new side-rosettes near the mother plant, but is rather limited in *P. officinalis*[29].

### Study sites and sampling design

**Figure 1:**
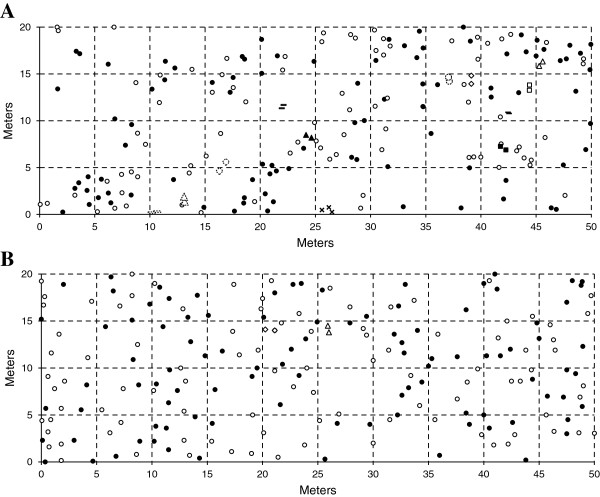
**Spatial distribution and corresponding morph type of 200 ramets per population.** Coordinates (m) and the corresponding morph type (black circles: L-morph, white circles: S-morph) of all sampled ramets in a plot of 50 x 20 m in **(A)** one Belgian population (Waardebroeken) and **(B)** one German population (Bertsdorf) of *P. officinalis*. Genetically identical ramets are indicated with different symbols (triangle, diamond, stripe, square, cross) in black or white depending on the morph type (L or S) to which the clones belong.

During the flowering season of 2012, four large (*N* > 1000) populations were sampled in two different parts of *P. officinalis*’ distribution range, separated by more than 800 km (Table 1). Two populations, Hofeberg (51% L-, 49% S-morphs) and Bertsdorf (53% L-, 47% S-morphs) were situated in eastern Germany, representing the core of the distribution area. Two populations, Kloosterbos (59% L-, 41% S-morphs) and Waardebroeken (60% L-, 40% S-morphs), were sampled in western Belgium, which is located beyond the western edge of the main distribution range [29,41]. This region only comprises twenty-six populations of *P. officinalis* and occupies an area of 17 × 10 km [28,29,33,35]. In each population, 200 ramets were randomly marked in a plot of 50 × 20 meters (Figure 1). For each marked ramet we recorded its position within the rectangle (X- and Y-coordinates) and the style-morph of the flowers (L- or S-morph). From each sampled ramet, young leaf material was collected and dried in silica gel for genetic analysis.

**Table 1:** **Extent of clonality, genetic diversity for each *P. officinalis *** **population and averaged for each region**

	**Population**	***N***	***G*/*N***	***A***	***G***	***H***_**O**_	***H***_**E**_	***F***_**IS**_
*Core*	Bertsdorf	5000	0.99	6.29	32.57	0.86	0.72	-0.21
	Hofeberg	1206	1.00	5.14	23.29	0.80	0.63	-0.27
*Disjunct*	Kloosterbos	4000	0.99	4.00	10.29	0.82	0.68	-0.20
	Waardebroeken	5000	0.93	4.57	12.57	0.82	0.67	-0.23
***Average core***			**1.00**	**5.72**	**27.93**	**0.83**	**0.68**	**-0.24**
***Average disjunct***			**0.96**	**4.29**	**11.43**	**0.82**	**0.68**	**-0.22**

*N,* number of ramets; G/N, genets-to-ramets ratio; *A*, allelic richness; *G*, genotypic richness; *H*_O_, observed heterozygosity; *H*_E_, expected heterozygosity under random chromosome segregation (Thrall and Young, 2000); *F*_IS_, inbreeding coefficient calculated as 1 - *H*_O_ / *H*_E_.

### Microsatellite analysis

DNA was extracted from 20 mg of silica-dried leaf tissue per sample following the Nucleospin Plant II protocol (Machery-Nagel, Düren, Germany). The polymerase chain reaction (PCR) was performed in the 2720 Thermal Cycler (Applied Biosystems, Foster City, California, USA) in a total volume of 10 μL containing 5 μL QIAGEN Multiplex PCR Master Mix (Hilden, Germany), 3 μL RNAse-free water, 1 μL template DNA and 1 μL of one of both multiplexed primer combinations presented by the Molecular Ecology Resources Primer Development Consortium et al. [46]. Both primer multiplexes had a thermocycler profile with initial denaturation of 15 minutes at 95°C; 25 cycles of 0.5 minutes at 95°C, 1.5 minutes at 59°C and 1 minute at 72°C, and a final elongation of 30 minutes at 60°C. Amplified microsatellite loci were sized on an ABI Prism, 3130 Genetic Analyzer (Applied Biosystems) in another 10 μL solution containing 1 μL of the PCR reaction, 8.8 μL formamide and 0.2 μL of the Applied Biosystems’ GeneScan 500 LIZ size standard and scored subsequently with GeneMapper® Software v4.0 (Applied Biosystems).

### Data analysis

#### Within-population genetic diversity and clonality

Although our sampling scheme was not specifically designed to detect genetically identical ramets, we compared the extent of clonal growth (genets-to-ramets ratio) between regions and between L- and S-morphs of all sampled populations. A matrix of mean (averaged across all loci) pairwise genetic distances among all individuals per population was obtained using the “meandistance.matrix” function in POLYSAT (version 1.2-1), a package in R [47] that was specifically designed for the analysis of polyploid microsatellite data when allele copy number is not known in partially heterozygous genotypes [48]. Clone mates were identified as combinations of ramets separated by a genetic distance of 0.

For each population, and based on the identified genets only, we calculated allelic richness (*A*), genotypic richness (*G*) (i.e. the number of genotypes averaged over all loci), observed (*H*_O_) and expected (*H*_E_) heterozygosity and the inbreeding coefficient (*F*_IS_) using the software AUTOTET [48]. AUTOTET was specifically designed for the analysis of autotetraploid genotypic data and computes the observed heterozygosity by weighting the five different classes of possible genotypes per locus (*AAAA*, *AAAB*, *AABB*, *AABC*, *ABCD*) inversely to the probability that any two of their alleles are identical by descent. Expected heterozygosity and inbreeding coefficient are calculated based on random chromosomal segregation (for details see [49]).

#### Spatial autocorrelation analysis

Clustering of floral morphs was studied using join count analyses for binary data (L-morph versus S-morph). Join count statistics (observed, expected value, standard deviation) were calculated for all the L-L joins and S-S joins under the assumption of 'nonfree’ sampling (= sampling without replacement) using PASSaGE 2 for each of the following distance classes: 1.5, 3, 5, 7, 10, 15, 20, 25, 30, 35, 40, 55 m [50,51]. *Z*-values were calculated to test for significant deviation of the observed amount of equal morph pairs within a distance class from a random distribution of morphs for each of these twelve distance classes and for both kind of joins (L-L/S-S), separately [51]. Significant clustering was statistically tested using a simple linear regression correlating the obtained *Z*-values per distance class and the logarithm of the distance of each class and calculating the regression slope *b*_Z_ per population for each morph separately.

To compare the extent of fine-scale spatial genetic structure between regions and morph types, we conducted spatial autocorrelation analyses using SPAGeDi 1.3, a software suited for analyzing autotetraploid microsatellite data [52]. Under isolation-by-distance processes, theory predicts that the multilocus kinship coefficient *F* decreases approximately linearly with the natural logarithm of the physical distance ln(r) between individuals [53]. The average multilocus kinship coefficients *F*_*ij*_[54] were computed and averaged for the same twelve distance classes as used in the join count analyses. To test the hypothesis that there was significant SGS, the observed regression slope of *F*_*ij*_ on ln(*r*_*ij*_), *b*_F_, was compared with those obtained after 10,000 random permutations of individuals among positions. This procedure has the advantage that all the information is contained in one single test statistic, and that the results are independent of an arbitrarily set of distance intervals [32]. The '*Sp*’ statistic, developed by Vekemans and Hardy [32] to compare SGS between species, was calculated as –*b*_F_/(1 - *F*_(1)_), where *F*_(1)_ is the average multilocus kinship coefficient of all ramet pairs belonging to the first distance interval. '*Sp*’ values were determined for each population with and without including clone mates (genetically identical ramets).

For each population, we also calculated regression slopes (*b*_F_) and *Sp* values for each morph separately, and for pairs of opposite morphs (between morph). A difference in spatial relatedness within L- and S-morphs provides insights into putative morph-specific mating differences, whereas a spatial genetic structure among pairs of opposite morphs provides information about gene dispersal distances. Strong between-morph SGS may reveal local pollen dispersal by insects transferring pollen mainly to flowers at close range [55]. Because the number of plants was insufficient in the smallest distance class (1.5 meters) to obtain at least 100 pairwise kinship coefficients [52], the first distance class in these analyses covered all distances up to 3 m.

Heterogeneity in SGS between populations was investigated by testing whether the decrease in relatedness with distance (*b*_F_) differed between pairs of populations using an analysis of covariance (ANCOVA) and adding the interaction term ln(*r*_*ij*_) × population. Kinship coefficients of the two populations within each region were averaged per distance interval and the average slope was compared between the regions in the same way. In addition, differences in SGS between the two morph types within each population were tested using a similar analysis of covariance (ANCOVA).

## Results

### Within-population genetic diversity and clonality

The disjunct populations had a total of 38 alleles (Kloosterbos: 28; Waardebroeken: 32) for all seven microsatellite loci whereas the two core populations had 48 alleles in total (Bertsdorf: 44; Hofeberg: 36). Overall allelic and genotypic richness were higher in core (*A*: 5.72, *G*: 27.93) than in disjunct populations (*A*: 4.29, *G*: 11.43). We found similar average values of observed, expected heterozygosity and inbreeding coefficient for the core and disjunct region (Table 1). Clonal growth was limited in the sampled populations with an average genets-to-ramets ratio (*G*/*N*) of 1.00 (= no clones detected) in the core region and 0.96 in the disjunct region (Figure 1). The Belgian population 'Waardebroeken’ had the lowest *G/N* ratio (0.93) due to 11 pairs and 1 trio of clone mates among the sampled ramets (Table 1, Figure 1A).

### Spatial autocorrelation analysis

**Figure 2:**
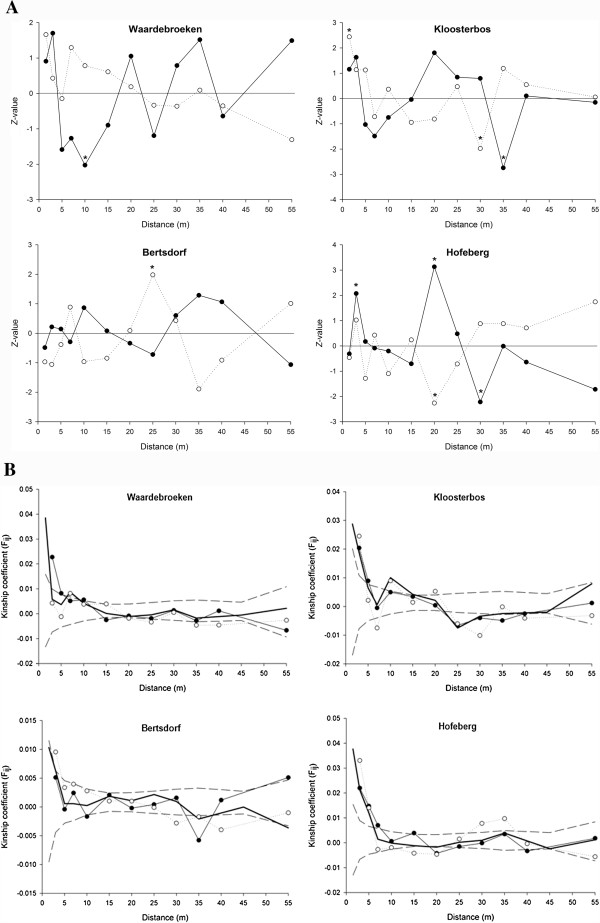
**Spatial autocorrelograms for two Belgian (Waardebroeken, Kloosterbos) and two German (Bertsdorf, Hofeberg) populations of *P. officinalis *of morph-joins and Nasons’s kinship coefficients. (A)** Results of the join count analyses for L-L (black circles) and S-S (white circles) joins, separately. Positive *Z*-values indicate higher aggregation whereas negative *Z*-values indicate negative spatial autocorrelation. Asterisks indicate significant deviations of random morph distribution (zero line) in a particular distance class. **(B)** Results of the genetic autocorrelation analyses. The solid line without symbols represents the correlogram for the entire population with the dashed lines indicating upper and lower 95% confidence intervals obtained after 10,000 permutations. Circles represent average multilocus kinship coefficients for the L-morph (black) and the S-morph (white), separately.

*Z*-values obtained from join count analyses (genets-only analysis) correlated to class distances (Figure 2A) showed no significant spatial autocorrelation in both morphs (*P* > 0.085) except for significantly decreasing clustering of the S-morph in the 'Waardebroeken’ population of the disjunct range (*b*_Z,L_ = 0.109, *P* = 0.783; *b*_Z,S_ = -0.554, *P* = 0.004). Figure 2A, however, indicates that positive spatial autocorrelation or clustering occurred at distances up to 5 meters in all populations except for the central 'Bertsdorf’ population (*b*_Z,L_ = 0.099, *P* = 0.640; *b*_Z,S_ = 0.281, *P* = 0.373). *Z*-values for higher distance classes were highly variable and showed no consistent decrease or increase ('Kloosterbos’: *b*_Z,L_ = -0.262, *P* = 0.496; *b*_Z,S_ = -0.554, *P* = 0.085; 'Hofeberg’: *b*_Z,L_ = -0.428, *P* = 0.295; *b*_Z,S_ = 0.308, *P* = 0.352), except for the S-morph of the population 'Waardebroeken’ (Figure 2A).

The spatial autocorrelation analysis revealed significant SGS in all four sites (Figure 2B, Table 2). The slope of the regression line between the kinship coefficient and the natural logarithm of the distance separating the genets (*b*_F_) varied between -0.0013 and -0.0059 (Table 2). The decrease in relatedness (genets-only analysis) was, however, significantly less steep in the 'Bertsdorf’ population from the core of the range as compared to the three other populations. The average decrease in relatedness was almost twice as large in populations of the disjunct region (*b*_F_ = -0.0051, *Sp* = 0.0053) compared to populations in the core of the range (*b*_F_ = -0.0028, *Sp* = 0.0030), but the extent of SGS did not differ significantly between regions (*P* = 0.48). The low SGS in the core and the high SGS in the disjunct range were mainly the result of the contrasting SGS in the 'Bertsdorf’ (*b*_F_ = -0.0013, *Sp* = 0.0014) and 'Kloosterbos’ (*b*_F_ = -0.0059, *Sp* = 0.0061) populations, respectively since SGS in the two remaining populations 'Hofeberg’ (*b*_F_ = -0.0043, *Sp* = 0.0045) and 'Waardebroeken’ (*b*_F_ = -0.0043, *Sp* = 0.0044) did not differ significantly (*P* = 0.58).

**Table 2:** Spatial genetic structure parameters for each population total, within both morph (L,S) and between pairs of the opposite morph (between)

***Core***					***Disjunct***				
***Population***	***Morph***	***b***_**F**_	***F***_***(1)***_	***Sp***	***Population***	***Morph***	***b***_**F**_	***F***_***(1)***_	***Sp***
Bertsdorf	L	-0.0007 NS	0.0051 NS	0.0007	Kloosterbos	L	-0.0062**	0.0204*	0.0063
	S	-0.0033**	0.0095 NS	0.0033		S	-0.0057**	0.0245*	0.0058
	between	-0.70007 NS	0.0070 NS	0.0007		between	-0.0059***	0.0187*	0.0060
	**Total**	-0.0013*	**0.0103 NS**	**0.0014**		**Total**	-0.0059***	0.0288*	**0.0061**
Hofeberg	L	-0.0062***	0.0220*	0.0063	Waardebroeken	L	-0.0068**	0.0227*	0.0070
	S	-0.0036*	0.0331*	0.0038		S	-0.0043*	0.0043NS	0.0044
	between	-0.0039***	0.0217*	0.0040		between	-0.0028*	0.0142*	0.0028
	**Total**	-0.0043***	0.0377*	**0.0045**		**Total**	-0.0043***	0.0385*	**0.0044**
**Average**		**-0.0028**	**0.0240**	**0.0030**			**-0.0051**	**0.0337**	**0n0053**

*b*F, the regression slope of the pairwise kinship coefficients (*F*_*ij*_) on the logarithm of the geographical distance ln(r_ij_) with significance level, the average kinship coefficient among neighbors F_(1)_ with significance level and the '*Sp*’ statistic calculated as –*b*_F_/(1 - F_(1)_).

*P < 0.05, **P < 0.01, ***P ≤ 0.001; NS, not significant.

Within populations, significant fine-scale genetic structure was found for both morphs and between morphs in the disjunct populations 'Kloosterbos’ (*b*_F,L_ = -0.0062, *Sp*_L_ = 0.0063; *b*_F,S_ = -0.0057, *Sp*_S_ = 0.0058; *b*_F,between_ = -0.0059, *Sp*_between_ = 0.0060) and 'Waardebroeken’ (*b*_F,L_ = -0.0068, *Sp*_L_ = 0.0070; *b*_F,S_ = -0.0043, *Sp*_S_ = 0.0044; *b*_F,between_ = -0.0028, *Sp*_between_ = 0.0028) with a significantly stronger SGS in the L-morph of the latter population (*P* = 0.029) (Table 2, Figure 2B). The core population 'Hofeberg’ showed a similar pattern as the disjunct 'Kloosterbos’ population with significant *Sp* values within (*b*_F,L_ = -0.0062, *Sp*_L_ = 0.0063; *b*_F,S_ = -0.0036, *Sp*_S_ = 0.0038) and between morphs (*b*_F,between_ = -0.0039, *Sp*_between_ = 0.0040), whereas for the other core population 'Bertsdorf’ (*Sp* = 0.0014) we only found a significant SGS for the S-morph (*b*_F,S_ = -0.0033, *Sp*_S_ = 0.0033), but not for the L-morph (*b*_F,L_ = -0.0007, *Sp*_L_ = 0.0007), nor between the morphs (*b*_F,between_ = -0.0007, *Sp*_between_ = 0.0007) (Table 2, Figure 2B). Hence, in the disjunct range, SGS in the L-morph was significantly stronger compared to the S-morph in one population ('Waardebroeken’) whereas the opposite pattern was found in the core 'Bertsdorf’ population (*P* = 0.018; Table 2).

## Discussion

Comparing genetic diversity and the extent of SGS between populations at a broad geographical scale allows gaining insights into the possible factors that influence (non-random) gene flow in the population such as mating system, pollinator community, population density and isolation. Consistent with previous results [29], we found lower within-population genetic diversity and a lower average allele number in disjunct *P. officinalis* populations (*A* = 4.29; *G* = 11.43; *N*_allele_ = 30) compared to core populations (*A* = 5.72; *G* = 27.93; *N*_allele_ = 40). This is in line with the many studies that have investigated patterns of genetic diversity across broad geographical scales (reviewed in [3]). On the other hand, there are very few studies that have compared SGS across a species’ range [56,57]. These studies reported high variability in spatial genetic structure between core and peripheral populations, either associated with a decrease in plant density [56] or with an increase in fragmentation/disturbance [57] towards the range edge. Peripheral populations of the mixed-mating conifer *Thuja occidentalis* exhibited on average a twofold higher SGS (*Sp* = 0.023) compared to core populations (*Sp* = 0.014) due to anthropogenic disturbances and population fragmentation at the periphery of the range (Nova Scotia) [57]. In the Sitka spruce, Gapare and Aitken [56] found significant SGS in ecologically peripheral and disjunct populations, whereas individuals were randomly distributed in continuous core populations.

Heterostylous plant species are characterized by a heteromorphic incompatibility system, which promotes disassortative/intermorph mating by preventing fertilization by own pollen and pollen from plants of the same style morph [58-61]. In theory, SGS in populations of heterostylous species is expected to be weak, as the obligate cross-pollination in self-incompatible species will contribute substantially to the overall gene dispersal within populations (σ^2^ = σ^2^_seed_ + ½*σ^2^_pollen_) [1,32]. In contrast, pollen flow in selfing species does not significantly contribute to gene dispersal [1,32,62]. The few studies on SGS in distylous species (mainly *Primula* species) have mostly found low *Sp* values, ranging from 0.0112 for *Primula cuneifolia*[63] and *P. vulgaris*, 0.0140 for *P. veris*[32], 0.010-0.0180 for *P. elatior*[64,65] to 0.0196 for the partially self-compatible *P. sieboldii* (*Sp* inferred as the inverse of the neighborhood size, *Nb* = 50.9) [32,66], indicating that spatial genetic structure is in general weak.

We found significant SGS in all *P. officinalis* populations, and even though the average SGS (*Sp* = 0.0042) was somewhat smaller than values found for *Primula* species, the *Sp* value was of the same order of magnitude as found for other self-incompatible, animal-pollinated herb species, e.g. *Arabidopsis halleri* (*Sp* = 0.0047) and *Lesquerella fendleri* (*Sp* = 0.0076) (reviewed in [32]). Interestingly, SGS was almost twice as high in the disjunct part of the distribution range (*Sp* = 0.0053) compared to the center of the range (*Sp* = 0.0030) although this contrast was mainly attributable to two contrasting populations located in the two regions. Adding more populations to our analysis would have increased the power of the statistical analysis and may have resulted in a stronger difference in SGS between disjunct and core populations. Unlike the heterostylous species *Decodon verticillatus*, in which clonal growth increased towards the northern edge of the range [67], we did not observe a shift to extensive clonal growth in *P. officinalis* to assure reproduction at the range edge (Table 1) [29]. As a result, clonal growth did not significantly contribute to the stronger genetic structure in disjunct populations.

Fine-scale genotype clustering in species with a genetically determined incompatibility system (S-locus) reduces the proximity of potential mates and may decrease local female reproductive success [68,69]. However, Brys and Jacquemyn [35] found that the dependence of potential mates was stronger in the S-morph, which showed a strong decrease in seed set with increasing distance to the opposite morph within the disjunct region of western Belgium. The L-morph, on the other hand, received much higher proportions of illegitimate pollen than the S-morph and seed set was less dependent on the distance to the opposite morph [33,35]. These results were explained by the fact that L-morph pollen of *P. officinalis* is deposited in significantly larger amounts on both the proboscises and heads of its principal pollinators compared to S-morph pollen. This was attributed to the limited length of the proboscis of these principal pollinators (mostly short-tongued bumblebees) in combination with the occurrence of floral hairs at the entrance of the corolla tube of L-morph flowers, which easily collect pollen as the proboscis moves past (see [33]). If short-tongued bumblebees force their heads into the corolla tube of L-morph flowers, their heads become easily contaminated with L-morph pollen grains that are deposited on these floral hairs. This phenomenon, in combination with the fact that L-morph flowers produce nearly twice as much pollen as S-morph flowers, explained the high rates of illegitimate pollination in the L-morph in the disjunct region [33].

Interestingly, the asymmetry in mating between L- and S-morphs found by Brys et al. [33] in the same region was translated into a significant difference in spatial genetic structure between the morphs in the disjunct 'Waardebroeken’ population (*b*_F,L_ = -0.0068 vs. *b*_F,S_ = -0.0043, *P* = 0.029), whereas no such difference was observed in the center of the range (*b*_F,L_ = *b*_F,S_ = 0.0035, *P* = 0.47) (Table 2). In *Primula veris*, another distylous species exhibiting weak self-incompatibility in the L-morph, Van Rossum and Triest [55] found higher relatedness among neighboring plants of the L-morph which they also explained by asymmetry in mating. In *P. elatior*, which exhibits a strict incompatibility system, however, the same authors did not find a significant difference in SGS between both morphs [65]. As in most distylous species the L-morph of *P. officinalis* represents the recessive (ss) genotype and the S-morph the heterozygous (Ss) genotype [70]. Mating among morphs due to weak incompatibility may therefore result in fine-scale clustering of the morphs especially in the L-morph and finally result in L-morph-biased populations [71]. However, we did not find significant clustering of the morphs in the sampled populations (except for the S-morph in one disjunct population). Nonetheless, the spatial autocorrelation analysis showed high *Z*-values and thus positive spatial autocorrelation at distances smaller than 5 meters except for the core population 'Bertsdorf’, which also had a significantly lower SGS compared to the other three populations (Figure 2A). However, it is likely that more intensive sampling and the mapping of all individuals within a given plot will provide better insights in the extent of fine-scale spatial clustering in natural populations. For example, in a dense Belgian population in which the position and morph type of each flowering individual were recorded within a 20 × 8 m plot significant spatial clustering was observed, particularly in plants of the L-morph [36].

## Conclusion

In this study, we documented substantial variation in the fine-scale SGS of a heterostylous species across a broad geographical scale. Although the extent of fine-scale SGS tended to be low, we showed that the extent of SGS was more than four times stronger in one of the disjunct populations than in one of the core populations. This difference in SGS between these populations can be most likely explained by differences in mating between regions in combination with population-specific characteristics. The mixed mating system of the L-morph as opposed to the outcrossing system of the S-morph was earlier found to influence the genetic structure of the metapopulation in the disjunct range of *P. officinalis*, due to lower levels of gene flow between the partially selfing L-morphs of distant populations [28]. These results were to some extent confirmed here as the extent of SGS was substantially higher in the L-morph than in the S-morph in the disjunct part of the range, where pollen flow within the L-morph is possible.

## Authors’ contributions

SM carried out the sampling in the field, the genetic analyses and drafted the manuscript. HJ directed the research, participated in its design and assisted together with OH, with the drafting of the manuscript. All authors read and approved the final manuscript.
